# Gastro-colic Fistula due to Acid Ingestion

**DOI:** 10.4021/gr2010.02.173e

**Published:** 2010-01-20

**Authors:** Atul Sachdev, Monica Gupta, Ashok Kumar Attri

**Affiliations:** a Department of Medicine, Government Medical College and Hospital Chandigarh, India; b Department of General Medicine, Government Medical College and Hospital Chandigarh, India; c Department of Surgery, Government Medical College and Hospital Chandigarh, India

**Keywords:** Corrosive, Fistula, Stomach, Colon

## Abstract

Corrosive ingestion can cause serious damage to the upper gastrointestinal tract. The acute consequences could range from mild erythema to gangrene and perforation. Delayed consequences could be strictures of the oesophagus presenting as dysphagia or gastric, pyloric and antral stenosis presenting as gastric outlet obstruction. Gastro-colic fistula as a complication of acid ingestion is a rare clinical entity. We report this case of a patient who presented with complaints of dysphagia and recurrent vomiting of foul smelling brownish vomitus two months following suicidal acid (sulphuric acid) ingestion. The presence of a gastro-colic fistula was confirmed with a barium meal examination.

## Introduction

Corrosive ingestion is a common cause of upper gastrointestinal tract injury in India. Esophageal burns, gastric perforation and peritonitis present with acute life threatening emergencies. However, it is the delayed complications of esophageal strictures and gastric outlet obstruction that lead to considerable morbidity. The present case here is highlighting an extremely uncommon delayed manifestation of acid ingestion in the form of gastro-colic fistula which to date has been scantly reported in literature.

## Case Report

A 45 years old unemployed man following a bout of alcohol binge presented to the hospital emergency with history of suicidal ingestion of approximately 100 ml of sulphuric acid. Examination of the oral cavity revealed superficial ulcers on the inner cheeks, the posterior pharyngeal wall and erythema of the epiglottis. There was no respiratory discomfort or hemodynamic instability. A chest X-ray and abdominal X-ray done to look for perforation was normal. After the initial resuscitation, he was taken up for upper gastrointestinal endoscopy which revealed deep ulcers of esophagus at approximately 20 cm from the incisors and scattered superficial ulcers in the rest of the esophagus along with diffuse erythema. There were deep ulcers in the antrum of the stomach extending to the pylorus along with ulcers in the mid body on the posterior surface. Duodenum was normal.

**Figure 1:**
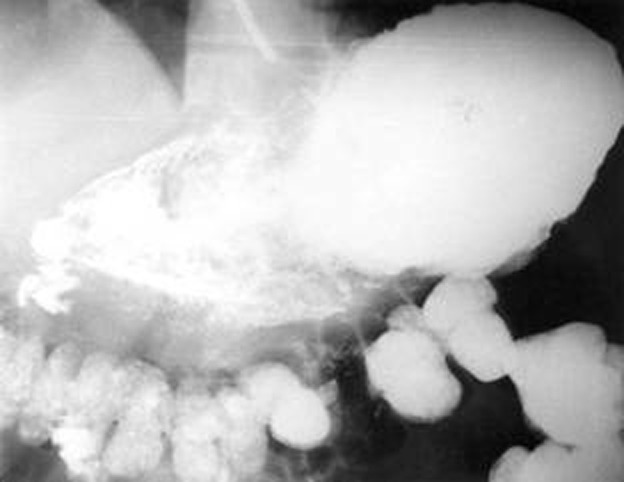
Barium meal examination showing the presence of gastro-colic fistula.

The patient was managed conservatively and discharged after 10 days of hospitalization. Two months after discharge he presented with complaints of dysphagia and recurrent vomiting of brownish foul smelling liquid. An upper gastrointestinal examination revealed a tight stricture at 20 cm, which did not allow the endoscope (Pentax videoscope EG 3840) to go beyond. A barium swallow and meal follow through examination showed the presence of a 3 cm long stricture in the upper esophagus. The rest of the esophagus was normal. The stomach showed slight lack of distensibility with slight thickening of the folds in the fundus. There was very little flow of barium into the duodenum, but instead the transverse colon showed opacification along with descending colon, suggesting a possibility of gastro-colic fistula in the proximal body of the stomach from the greater curvature (Fig. 1).

Subsequent upper gastrointestinal endoscopy of the stomach done after esophageal dilatation with Savary Gilliard dilators revealed severe antral and pyloric stenosis with scarring. The endoscope could not be negotiated into the duodenum. No definite gastro-colic opening could be appreciated. A colonoscopic examination done to look for any abnormal opening in the colon also revealed no abnormal opening.

A feeding jejunostomy was done and patient was planned for a second stage definitive surgery. Laparatomy was done three months later. The procedure performed was distal gastrectomy along with Billroth 1. Operative findings were pyloric stenosis, scarring and adhesions between the stomach and colon. The scarred site was probably the site of fistula, which had healed. Subsequently the feeding jejunostomy was removed. The patient is doing well and is gaining weight.

## Discussion

Caustic ingestion can produce serious injury to the upper gastrointestinal tract [1]. The injury could be due to ingestion of an acid or an alkali [2, 3]. In India, we see large number cases of acid ingestion, which is mostly from a suicidal intent. Caustic ingestion can present with visible injury to the lips, oral cavity and pharynx. Symptoms may include persistent salivation, vomiting, hemetemesis, melena, dysphagia, odynophagia, and chest or epigastric pain [4-6]. There may be hoarseness and respiratory stridor as well. Some patients may present with features suggestive of mediastinitis like dyspnoea, stridor or shock. Gastric perforation may also occur and present as acute peritonitis [1]. These acute symptoms generally settle with conservative management but may be progressive. Delayed manifestations occur in the form of dysphagia as a result of esophageal stricture or features of early satiety, gastric outlet obstruction and progressive weight loss which generally come after 3 - 8 weeks of corrosive ingestion [3, 7].

Fistula between various abdominal organs following corrosive ingestion has been extremely rarely reported. A pancreaticocolic fistula has been seen at surgery, which was done in a patient having extensive burns of the upper gastrointestinal tract following acid ingestion [8]. Gastro-colic fistula as a result of corrosive ingestion has been rarely described in the literature. We report the occurrence of such a complication following acid ingestion. It manifested as a delayed complication two months after acid (sulphuric acid) ingestion. Possibly this complication occurred at the time of acute injury but did not manifest earlier. Only when the gastric antrum and pyloric region got narrowed and patient started having features of gastric outlet obstruction, it manifested because of increased gastric contents and pressure. This is a very unusual complication of corrosive ingestion and should be kept in mind in case a patient comes back with recurrent vomiting of foul smelling brownish material.
